# Caribbean red snapper fishing performance indicators in Brazilian amazon shelf: Is it the beginning of the end of a fishing system?

**DOI:** 10.1371/journal.pone.0300820

**Published:** 2024-05-01

**Authors:** Niedja Mescouto, Ualerson Iran Peixoto, Diego Gomes Trindade, Hanna Moura, Bianca Bentes

**Affiliations:** Postgraduate Program in Aquatic Ecology and Fisheries, Center for Aquatic Ecology and Fisheries in the Amazon, Laboratory of Fisheries Biology and Management of Aquatic Resources, Group for Ecology and Fisheries Management in the Amazon, Federal University of Pará, Belém, Pará, Brazil; Wroclaw University of Environmental and Life Sciences: Uniwersytet Przyrodniczy we Wroclawiu, POLAND

## Abstract

Red snapper fishing (*Lutjanus purpureus*) is an important fishing activity for the Brazilian economy due to its export. The scarcity of up-to-date information on this system’s ecology, economy, and social characteristics contributes to inefficient management. We analyze whether the commercial snapper fishery on the Amazon continental shelf is socioecologically sustainable. For this, an assessment tool was used that can be applied to fishing systems with little data, the Fisheries Performance Indicators (FPI). The results showed that the critical points of this activity are mainly related to the Ecological indicator (2.3) and the Economic indicator (2.8). The best indicator was the Community (3.8). The problems that put at risk the permanence of the activity and its maintenance are: (i) fishing for juveniles; (ii) illegal vessels; (iii) lack of collaboration of the fishing sector with science, and (iv) unreliability of data supplied. All the points mentioned make the snapper fishery on the north coast of Brazil socio-ecologically unsustainable in the long term.

## 1. Introduction

The depletion of fish stocks worldwide and concerns about overexploitation have fueled an ongoing debate about the current state and prospects of global fisheries, associated threats to marine biodiversity, and declining yields Available in for human consumption [1]. In the context of global fisheries, Lutjanids, also commonly known as snapper, have been the target of studies that demonstrate the vulnerability of this taxa and the traditionality of their catches, associated with economic and cultural issues [2, 3]. Currently, management approaches have aimed to reduce fishing pressure and recover depleted stocks to biomass and exploitation rates that allow maximum sustainable yield [4, 5].

Fishing targeting Lutjanidae has resulted in overfishing of this group in several countries, particularly in areas where the resource is highly exploited, such as in the United States [6], Mexico [7, 8], Indonesia [9], and Brazil [10], where there is a lack of specific management systems or regulations (fishing licenses, catch quotas, size limits, closed seasons) that contribute to increased exploitation of these resources [8]. In contrast, countries that have implemented management measures, such as the USA, have achieved positive results in rebuilding snapper stocks [11].

In Brazil, fishing targeting the species *Lutjanus purpureus* is of great economic importance due to its high commercial value and the high demand from restaurants and consumers [12, 13]. Additionally, snappers play a crucial role in marine ecosystems as population-regulating predators [14], contributing to ecological balance and serving as an important reef fishery resource [15, 16].

In the context of the red snapper fishery, the management has been one of the major challenges in the context of marine conservation and sustainability of human activities [17]. The scarcity of information is considered standard, especially in countries where there is no integrated monitoring system for this sector [18]. Even in fisheries of commercial interest, such as the red snapper, there is no availability of biological data (age, growth, and reproduction) or fisheries data (catches, effort and yields) as a subsidy for adequate management, which hinders a thorough assessment of the impact that fishing has been causing at the biological, economic and social levels. Since fishing is a multidisciplinary activity involving different dimensions, in recent years, some methodologies have been developed for a better understanding of environmental and socioeconomic characteristics in fisheries analyses [19–22].

With this, the concept of ecosystem-based management (EBFM) has become increasingly important, as it provides a holistic look at fisheries, considering different ecosystem elements and different dimensions beyond ecological ones, such as economic and social dimensions [23]. The EBFM concept and the need for more interdisciplinary approaches in fisheries science that consider social, economic, and environmental systems can assist in the development of more efficient management measures and more sustainable management practices [23].

One of the methodologies that seek to integrate the different dimensions of fishing into a single tool is the Fishery Performance Indicators (FPI) [24, 25]. The FPI is a rapid and low-cost assessment tool that can describe the state of fishing systems through a set of different metrics grouped into different dimensions, including stock, fishing, and post-capture state. It also includes three sustainability indicators: ecology, economy, and community—the Triple Bottom Line (TBL) [25]. Due to its flexibility, this tool has been used to examine fishery performance at global scales [26], can be applied in specific regions [27] and countries [28]. Furthermore, it can be effectively used in multi-species and single-species fisheries [29–31], in fisheries of developing countries [32–34], data-poor fisheries [35], investment projects [36], and can address, for example, the characteristics for effective management considering the three sectors of analysis [33, 37].

Analyses that integrate ecological, economic, and social dimensions can provide important information for management and interventions compatible with local realities can increase the sustainability of the fishery. Thus, the present study aimed to evaluate the commercial red snapper fishery using FPI tools in an integrated manner. This fishery was chosen because of its socioeconomic relevance and because the species, *L*. *purpureus*, lacks answers that clarify what the current situation of the fishery is, involving all its actors. This is the first attempt to study the snapper fishery in a comprehensive and holistic way, incorporating environmental, economic, and social aspects of the fishery. The results can be compared with other fishing systems worldwide, helping to formulate new recommendations for management measures.

### 1.1. Snapper fishing in Brazil

Red snapper exploitation is one of Brazil’s most profitable fishing activities, occurring mainly in the North and Northeast regions [38, 39]. The records of landings of this activity began in 1960 in the Northeast of Brazil [40, 41], later migrating to the North in 1982 after the depletion of stocks in that region. Currently, fishing still takes place throughout the Brazilian North and Northeast, with greater intensity in the North, where fishing is consolidated [20, 42].

**Fig 1:**
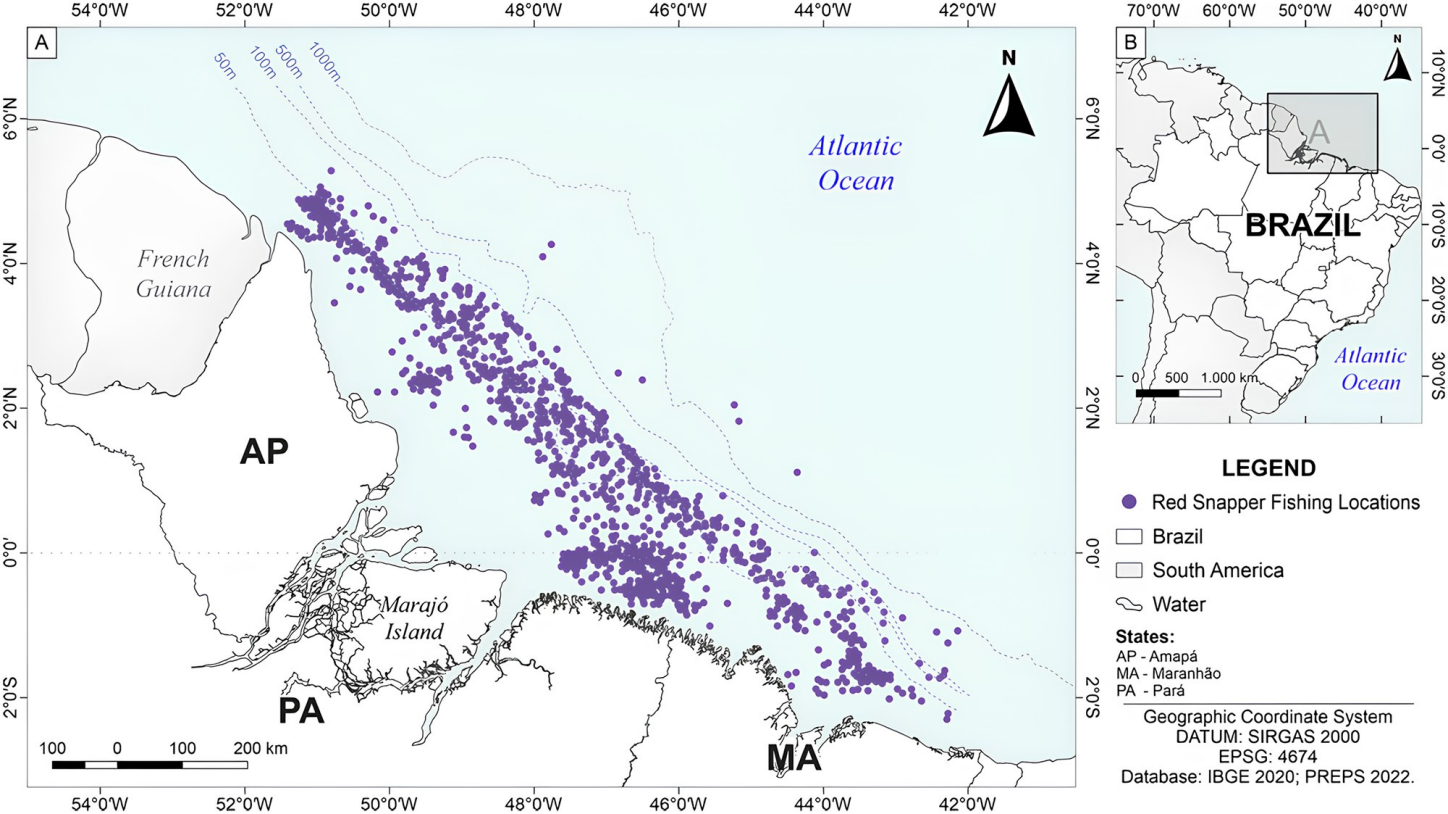
Commercial fishing area for the pargo *Lutjanus purpureus* (purple points) on the northern coast of Brazil. For more information about red snapper fishing on the Brazilian coast, visit: https://www.gov.br/mpa/pt-br/assuntos/cadastro-registro-e-monitoramento/monitoramento-e-ordenamento-da-pesca-do-pargo/monitoramento-e-ordenamento-da-pesca-do-pargo-2021-. Access date: 22/11/2023. Merely informative and unofficial content.

The fishing grounds of the Amazon continental shelf snapper fleet extend from Cabo Orange in Amapá State (51° W) to São Marcos Bay in Maranhão (46° W). The area is divided into two sedimentary basins, the Amazon Delta and the Pará-Maranhão basin [43] (Fig 1). In this area, there is an extensive region of rhodolith reefs (about 56,000 km) whose dimensions have recently been estimated [44, 45] and which harbour several endemic species [45]. The area is part of the Northern Brazil Large Marine Ecosystem (NBLME) and is highly influenced by the flow of the Amazon and Tocantins rivers, creating a highly dynamic and productive environment [46–50].

Red snapper fishing is carried out along the Brazilian northern coast during the period from March 1st to December 14th [60] by medium-sized boats, with wooden hulls ranging from 12 to 15m in length [42, 51]. The vessels are equipped with navigation aids (GPS and compass), communication devices (SSB and VHF radio), and sensing equipment (sonar). The crew consists of 7 to 12 fishermen, and the boats have a range of up to 30 days at sea [12, 51], capable of storing from 17 to 44 tons of fish and ice [52]. The main fishing gears used are the *’manzuá’* or *’covo’* (traps) and the *’pargueira* line’ (longline) [12, 20, 51].

**Fig 2:**
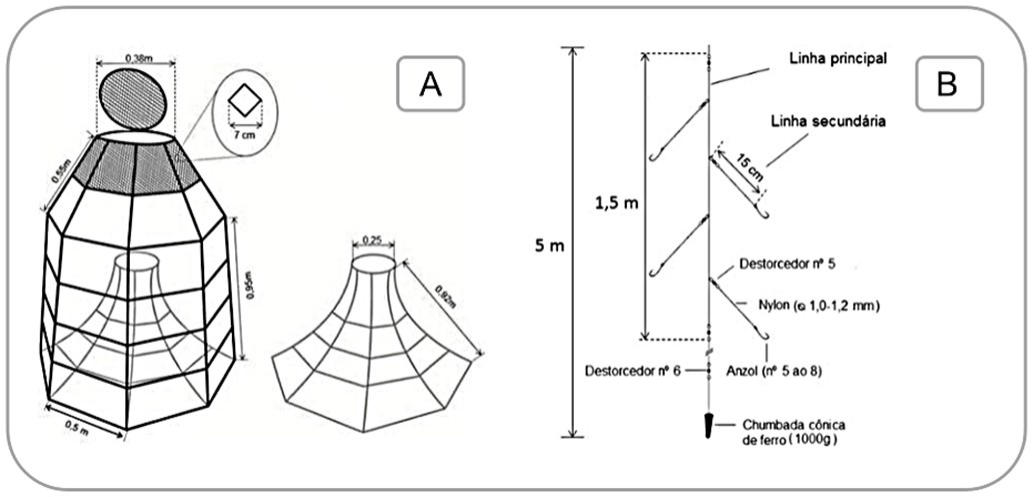
Schematic drawings of the fishing gears used in the fishing of the lane snapper *Lutjanus purpureus* on the northern coast of Brazil: (A) ’*manzuá*’ 1—escape door, 2—larger base, 3—smaller base; (B) *’pargueira* line’ : [12, 13].

The ’*manzuá*’ comprises octagonal iron traps covered with nylon^®^ mesh with 13cm spacing between opposite nodes, measuring 152 cm in length with an opening at the base, with each vessel carrying between 30 to 40 of these traps (Fig 2A) [13]. Conversely, ’*pargueiras* lines’ are vertical longlines consisting of a mainline of polyamide monofilament No. 200, which can vary from 200 to 400 m in length, to which the vertical longline is attached, with the number and length of secondary lines depending on the depth and speed at which the vessel operates (Fig 2B) [42]. Each mainline of the *’pargueiras* lines’ contains 20 to 30 hooks, ranging from No. 5 to 8 [42, 51], which are modified by fishermen from "J" to "C" (Fig 2C) shape to facilitate unhooking and baited with pieces of sardines or fish caught as bycatch [42, 51].

Among the two fishing gears mentioned, the ‘*pargueira* line’ has a greater selectivity due to the hooks used, but due to changes in pressure and movement during its hoisting, it can cause injuries to the captured specimens, which decreases the quality and consequently the sale value of the product [51]. Studies indicate that hooks with openings smaller than 1.6 cm capture a higher percentage of juvenile lane snappers; however, to protect juvenile stocks, the use of hooks with a 2.04 cm opening is recommended [53]. Currently, there are more vessels equipped with *’pargueira* line’ than with *’manzuá’* [42], probably because it is a less expensive and easier to maintain fishing gear.

Red snapper fishing holds significant social and economic importance for the Northern region of Brazil, generating substantial annual economic resources through exports [13]. Brazil’s red snapper exports primarily consist (97%) of whole eviscerated fish, with the United States (EUA) being the main destination market, absorbing between 80% and 95% of the production [54]. Exports exceeded $100 million between 2020 and 2022 [55–57]. However, the status of *L*. *purpureus* stocks in the Northern region remains unknown. In 2014, the species was classified as vulnerable (VU) on the national list of threatened species and has remained in the same category to the present year [58, 59].

Currently, the main mechanism for regulating the snapper fishery in force (Portaria MMA N 228/2018) determines the release of 150 fishing licenses and a closed season that occurs from December 15 to April 30, with a fishing area that extends from the north of the state of Amapá (in northern Brazil) to the limits of the states of Alagoas and Sergipe (northeastern Brazil), in depths above the 50 m isobath. Vessels must be registered and have satellite tracking equipment (National Program for Tracking Fishing Vessels by Satellite—PREPS) in addition to delivering onboard maps containing information about the ports of departure and arrival, activity data (number of hauls, location of the vessel, date, and time of fishing), and catch data (weight and species caught) on each trip [60].

## 2. Material and methods

### 2.1. FPI method and data source

**Fig 3:**
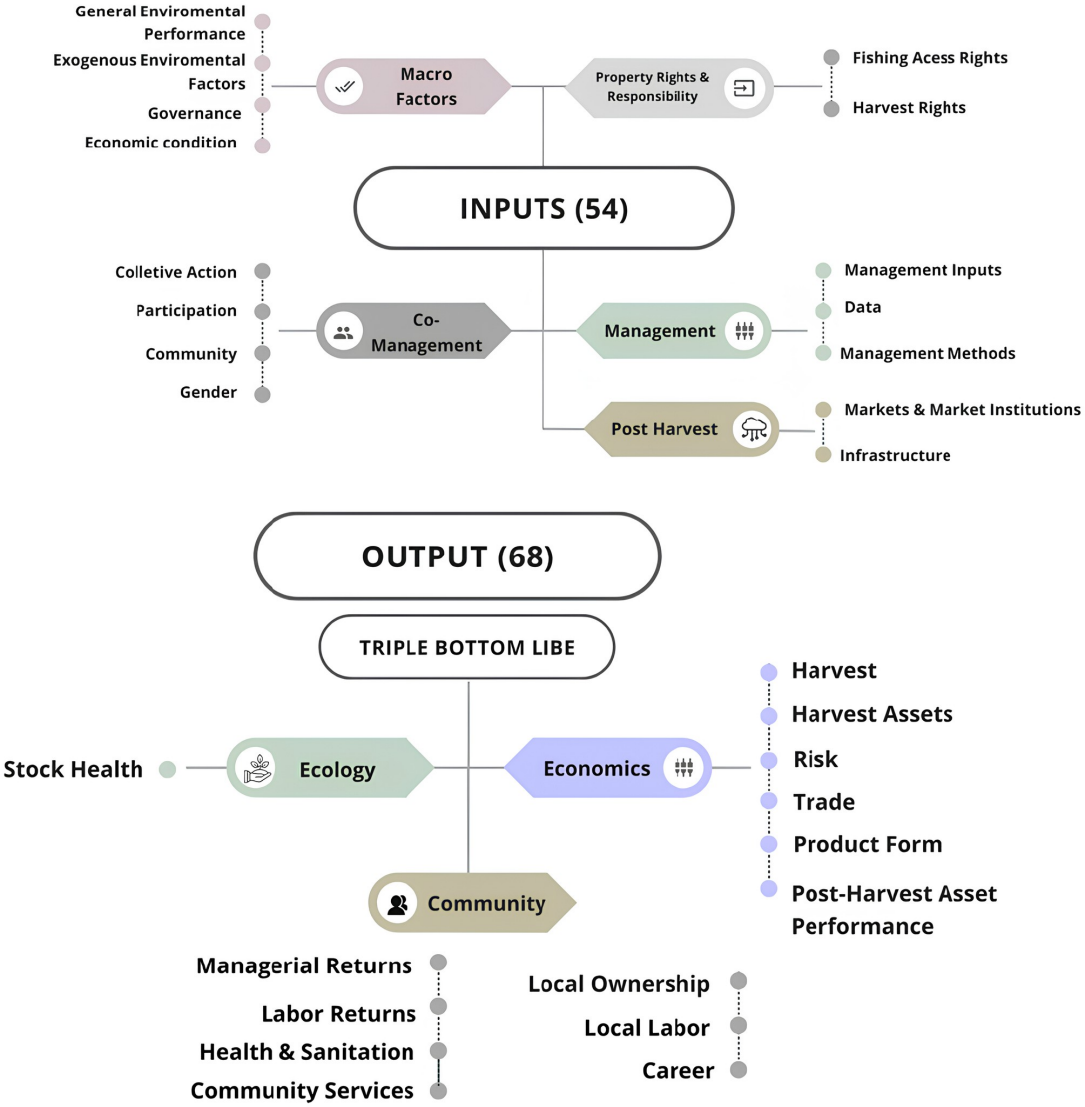
Schematic illustration of the metrics evaluated in the FPI methodology (fisheries performance indicators) for the red snapper fishery—*Lutjanus purpureus*—Of the Amazon continental shelf. Source: [34].

FPI, a tool developed [24], assesses fishery performance through 122 metrics that are fitted into dimensions, or issues of interest, to evaluate a fishery production system. The dimensions are classified into two major categories, named *input* and *output*. The *Output* category consists of 14 dimensions and 68 metrics distributed among ecological, economic, and community (TBL) indicators [24, 25]. The metrics are scored according to each indicator (Fig 3) and answer whether the fishery is generating sustainable socioeconomic and ecological outcomes. In this study, sustainability was defined as the ability to improve social and environmental performance to meet present needs without compromising the ability of future generations to meet their own needs [61].

The Input group is classified into 5 components, which contain fifteen dimensions, into which 54 metrics fit. These represent the existing conditions, environmental or otherwise, to produce good performance of the activity.

The input and output metrics were assigned values ranging from 1 (worst performance) to 5 (best performance) [24]. In addition, the metrics were evaluated according to the degree of reliability of the source of information, which ranged from A (accurate information: published data) to C (inaccurate information: data not officially published, coming from sources involved with fishing). In this way it was possible to weight the degree of uncertainty of the results. The average scores of the set of metrics determined the value of each dimension, and the averages of the dimensions determined the value of the component or indicator, ensuring robustness of the metrics even when little information was Available in.

The data were added into Microsoft Excel^®^ spreadsheets previously programmed to estimate averages, as well as to diagram radar graphs that summarized the scores (S1 and S2 Tables). The dimension values were compared to the mean scores found for 97 developing country (DC) fisheries and to the scores of the 10 best performing countries (T10) in the FPI method (*Iceland Nephrops lobster*, *Icelandic cod*, *Australia Western zone abalone*, *US-Alaska pollock*, *Japan wagu lobster*, *Australia Southern zone rock lobster*, *Japan Ofunato set-net salmon*, *Australia Spencer Gulf prawn*, *Norways purse seine and Japan Toyama Bay*). These reference scores were obtained with the collaboration of the research group that developed this method at the Institute for Sustainable Food Systems at the University of Florida and from Available in literature [24, 29].

The economic, social, and ecological data were extracted from the database provided by the Fisheries Improvement Projects—FIP Pargo, which operated during the years 2016 to 2018, in partnership with fishing entrepreneurs and the former Secretariat of Aquaculture and Fisheries (SAP) of the Federal Government. The FIP Pargo project collected data of biological character, related to the ecology and habitat of the snapper in the cities of Bragança and Augusto Corrêa, in addition, a socioeconomic and environmental survey was conducted through interviews with fishermen, entrepreneurs and other actors in the production chain of this resource.

## 3. Results and discussion

The percentage of the 122 metrics analyzed, according to information quality, was approximately 37% type A, 57% type B, and 4% type C (S1 and S2 Tables).

### 3.1. Input

**Fig 4:**
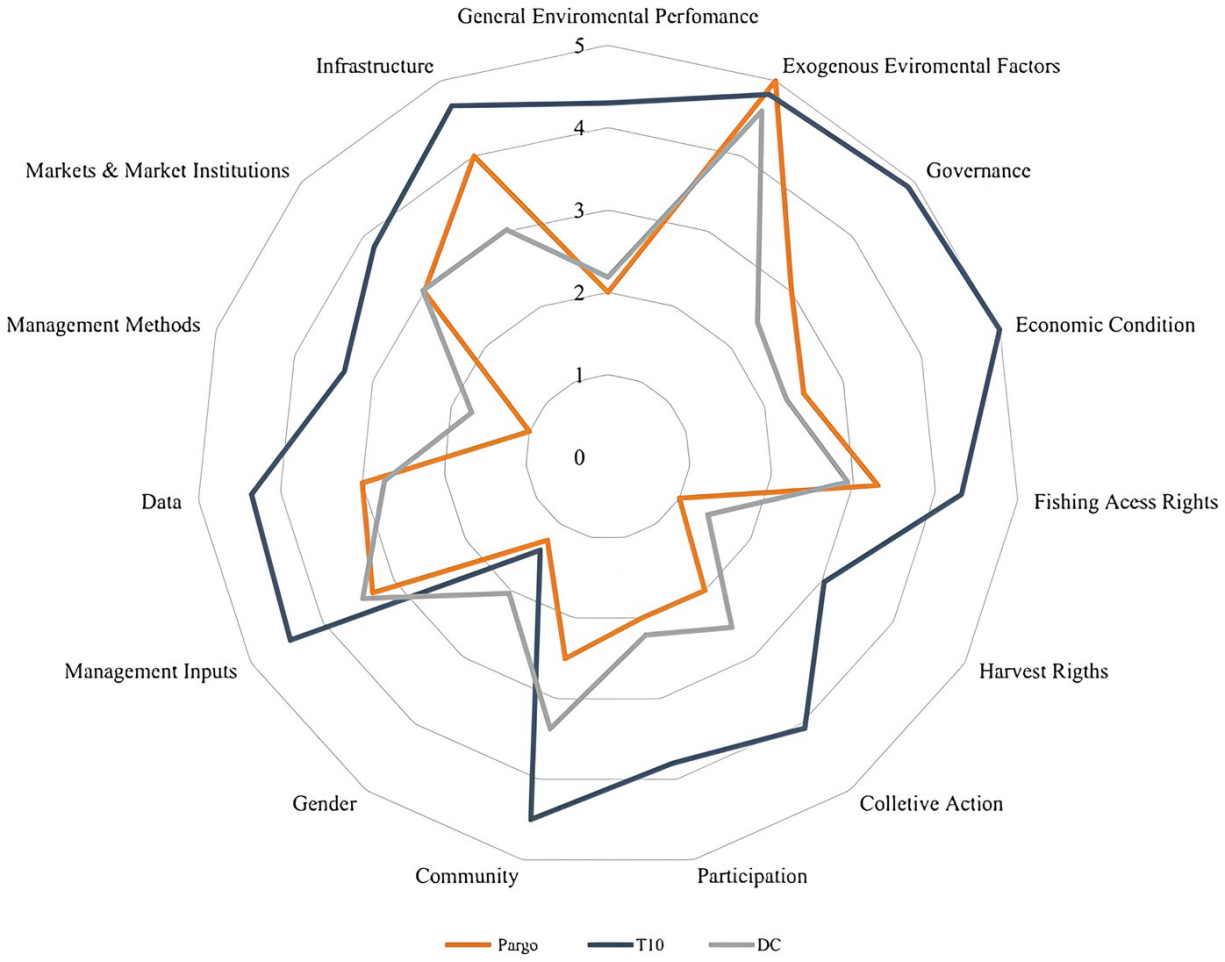
Average *Input* component scores of the large-scale artisanal snapper fishery *Lutjanus purpureus* (red) compared to the averages of the top 10 world fisheries (T10—blue) and the 97 Developing Countries (DC—gray).

**Fig 5:**
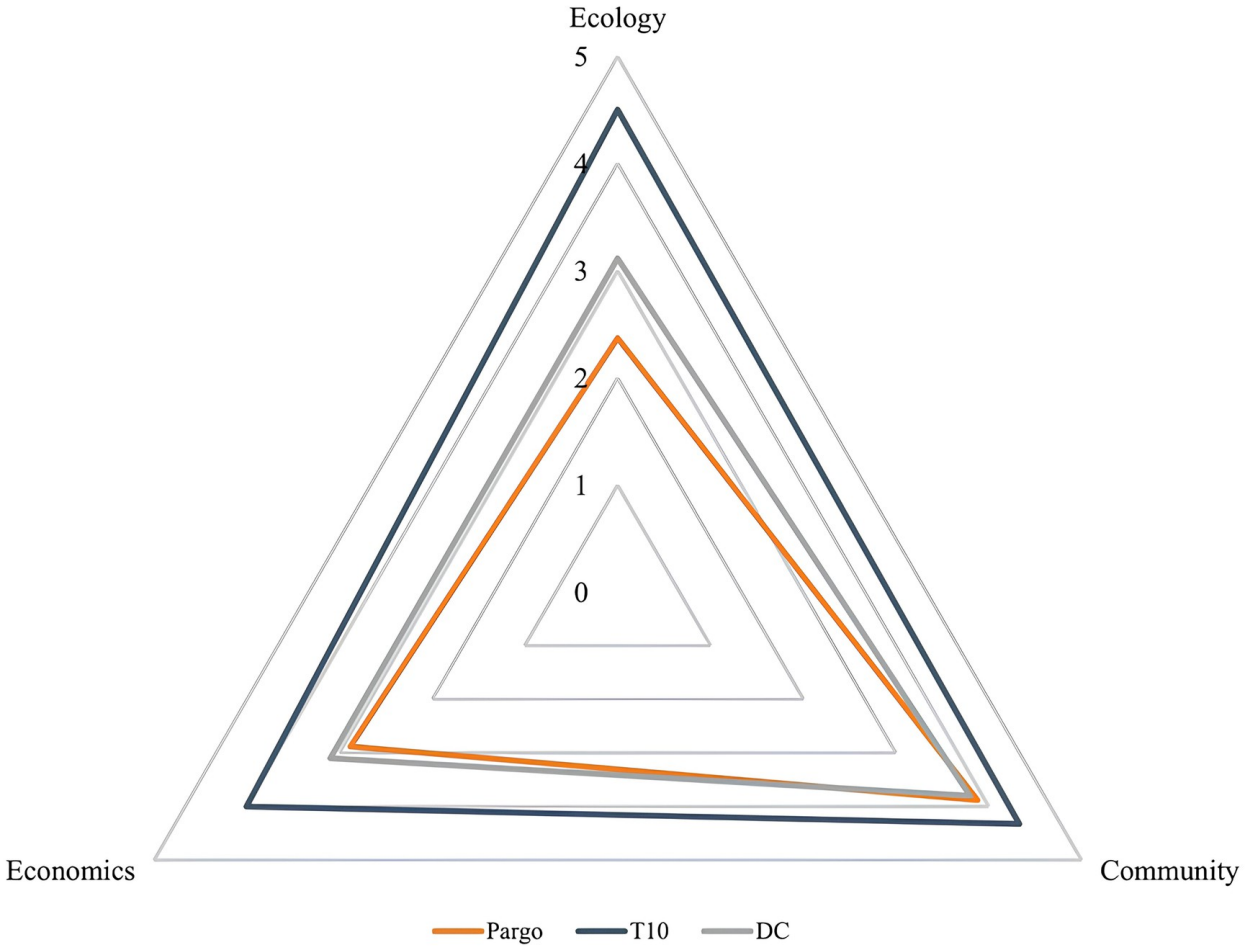
Average scores of *Inputs* dimensions of the large-scale artisanal red snapper *fishery Lutjanus purpureus* (red) compared to the averages of the top 10 world fisheries (T10—blue) and the 97 Developed Countries (DC—gray).

Input indicators were structured into 5 components containing 15 dimensions and 54 metrics (Fig 3). Overall, the average of the input indicators in this study was equivalent to that of developing countries denoting that the snapper fishery in Brazil does not differ from the performance of other fisheries in these countries. The components with the highest scores were *Post-Catch* (3.6) and *Macro factors* (3.1) and the others scored as low performers: *Management* (2.5), *Ownership and responsibility rights* (2.1) and *Co-management* (1.9) (Fig 4). Metrics such as *Management Methods* and *Data availability* among others are like developing country fisheries performance (Fig 5).

#### 3.1.1. Macro factors

The Macro Factors component evaluates the institutional state of the region. The score of this component (3.1) is justified by the low records of natural phenomena that cause significant structural changes in cities and the environment, such as hurricanes and typhoons. However, in 2022, Brazil dropped from the 55th position it held in 2020 to the 81st position in the overall ranking of the Environmental Performance Index (EPI), which assessed 180 countries in 2022 [62, 63]. Although Brazil ranked 81st overall, it placed significantly lower in specific areas: species habitat index (142nd), ecosystem services (142nd), and climate policy (133rd) [63]. Regarding fisheries, the EPI scores from 0 (worst performance) to 100, with 100 indicating that no fishery products from a particular country come from overexploited or depleted stocks [63]. For fisheries, Brazil ranked 53rd, and fish stock status at 38th [63]. This ranking, conducted by Columbia and Yale Universities, showcases how countries are addressing improvements in health and the environment, progressing in ecosystem protection, and mitigating climate change. These data, combined with those from the present study, can serve as a basis for government and non-governmental actions, as well as guide investors interested in doing business in the country.

The effects of climate change, which result in the global decline of biomass and changes in distribution patterns, diversity, and reproduction [64, 65] of aquatic resources [66, 67], are factors that have raised concerns in the Amazon region. The increase in temperature leads to changes in precipitation patterns and prolonged dry periods, reducing water discharge and the consequent productivity of the Amazon River [68]. These alterations are likely to favor aquatic species with high thermal tolerance [68]. Recent studies have found that elevated temperatures in coral reef regions can accelerate the growth of Lutjanidae larvae, but survival rates are lower [69].

Fishing activities in coastal regions face significant challenges due to global warming [70]. Fluctuations in sea surface height, associated with events like El Niño and La Niña, impact ocean circulation and coastal dynamics, influencing the migration and reproduction of the red snapper [71]. However, a more in-depth analysis of the role of climatic and oceanographic factors in the populations of Lutjanidae and other reef species in the Equatorial Atlantic is crucial [72].

#### 3.1.2. Property rights and responsibilities

The component obtained a low score (2.1) due to the scoring of its two dimensions: Fishing access rights (3.3) and Fishing rights (1.0). The *Property Rights and Responsibilities* component assesses the level and nature of control individuals can exert over the resource, along with the opportunities Available in to establish and safeguard well-designed ecosystem-based resource management structures.

In Brazil, snapper vessels are legalized through a fishing license that grants access to the catch [60], however, the absence of catch quotas or limits hinders the negotiation power among fishing stakeholders, hindering incentives for conservation and optimizing economic gains. Studies affirm that the adoption of catch limits is a good strategy for recovering declining populations [73]. The implementation of annual catch quotas in fisheries has generated discussions and positive outcomes for the recovery of stocks of species important to both artisanal [74, 75] and industrial [76] fishing sectors. In developed countries like the USA, where both large-scale artisanal and industrial fishing are controlled by catch limits, the species Lutjanus campechanus has been able to recover its stock [11, 77]. In Brazil, most fisheries lack catch limits and instead rely on management measures such as closed seasons and fishing licenses. The only fishery with a catch quota is for mullet (Mugil sp), which takes into account regular population assessments and has shown good performance in stock recovery [75].

In the case of snapper, there are estimates of maximum sustainable catch; however, they are flawed due to the lack of systematic production data [10]. A study states that snapper catches exceeding 4,500 tons are at risk of overfishing and biological unsustainability [73]. In the Snapper Recovery Plan, the authors also suggest the inclusion of an annual catch limit that aligns with an assessment of annual catches in conjunction with the maximum size of the fishing fleet [13]. We believe that a comprehensive and reliable assessment of snapper stocks in Brazil could be achieved if the recovery plan were implemented.

#### 3.1.3. Co-management

The *Co-management* component measures the role played by local actors in determining fisheries management. This component obtained a low score (2) due to the lack of representation of the agents of the productive chain (professionals in general from the fishing sector) in the decision-making process that involves management. The performance of this sector in decision making meetings is minimal, which justifies the low score obtained in the *Participation* dimension (2.0). Therefore, the fisher class does not have an active and influential representation in the management policies, and the decisions are almost always taken by the owners of the processing companies and the owners of the largest number of boats. The *Gender* component also got an unsatisfactory score (1.2). The fishing system is predominantly male, and there are no women in any of the decisive stages of the fishery, or post-catch. However, there are women in the processing industry, *Labor Participation in the Post-Fishing Sector* (2.0), even if in smaller numbers than men. The metric *Business Management Influence* and *Resource Management Influence* also received a score of 2.0 because the sector does not have equivalence of positions held by men and women, with men being more representative. On the other hand, women have been occupying important government positions in fisheries planning in Brazil and, in addition, there are female fisheries scientists, denoting increased female participation in the processes that guide fishing activity [78, 79].

Gender equality is a fundamental principle of good environmental governance and sustainable development [80]. Although women and men participate in the entire fisheries value chain [81, 82], gender differences are often more prominent compared to other sectors [83], with women being widely recognized only for ancillary roles, those in support of fishing activities performed by men [84].

#### 3.1.4. Management

The *Management* component assesses how well the management system functions in collecting information from both science and stakeholders and integrating it into policymaking. This dimension obtained an intermediate score (3.1). The dimension that contributed the most to this value was the *Management Inputs* (3.5), as in the case of red snapper fishing, the government enforces restrictions when a vessel fails to provide its logbooks, for example [60].

In Brazil, effort control has always been a central strategy in fisheries management. Thus, red snapper fishing is primarily regulated by Interministerial Ordinance SEAP-PR/MMA No. 42, dated 27/07/2018, which establishes the fishing limit from the northern border of the state of Amapá to the states of Alagoas and Sergipe (São Francisco River mouth), starting from the 50 m isobath. This regulation also includes closed seasons, which are in effect from December 15 to April 30, within the fishing limits established in the regulation [60]. However, it is recognized by decision-makers that landing activities still occur even during the closed season.

The *Data* dimension obtained an average score (3.0), slightly above the averages of fisheries in developing countries and below T1. Red snapper fisheries suffer from a common problem in Brazil: lack of reliable data on fishery production. The scarcity of periodic socioeconomic information and stock status information is a major problem for resource implementation and quantitative control for fisheries management [85]. Although the FAO report [81] indicates a stable situation for the species during the last 20 years, some research warns about the possibility of overfishing due to the reduction in the size of captured individuals, age and weight in the samples collected [12, 13, 86, 87]. Even in fisheries of commercial interest, such as that of snapper, data, whether biological (age, growth and reproduction) or fisheries (catches, effort and yields) are insufficient for the projection of future scenarios even through data poor methods [88].

The *Management Methods* dimension had the worst score (1.0), below the average for DC (1.7) (Fig 5). All metrics included in this dimension (*MPAs and sanctuaries*, *Spatial Management*, *Fishing Mortality Limits*) had scores considered insufficient, because the red snapper fishery, even with spatial control of the 50 m isobath, occurs in a marine area without fisheries monitoring (either by enforcement or remotely) that promotes species conservation and data collection to allow periodic estimates of fishing mortality and stock status. Thus, as there is no effective monitoring of catches (even though we have a flawed mapping system) and satellite monitoring of vessels, there are no parameters that can substantiate limits or reference points.

#### 3.1.5. Post-Harvest

The Post-Harvest dimension assesses the marketing conditions of fishery products and obtained an average score of 3.6. The Markets and Market Institutions metric scored 3.3 due to North Coast snapper being predominantly destined for the international market (Bentes et al., 2017) with established buyers, which directly contributed to the low scores assigned to the Landings Pricing System (2.0) and Buyer Numbers metrics (2.0). The landing pricing system is important for both fishermen, as it directly affects their income, and for buyers and seafood traders who utilize this information to determine prices for the end consumers.

This behavior is common and found in other activities where the target resources are export commodities [34]. However, it is recognized that a small percentage of the production is directed to the domestic market, particularly specimens that do not fit the "export type" (which requires snappers weighing less than 900g, among other sanitary requirements). Notably, the current production of red snapper is primarily aimed at the Asian market, which is considered less demanding than the European and North American markets [56].

The *Infrastructure* dimension obtained a good score (4) similar to the top T10 world fisheries and other large-scale artisanal and industrial fisheries of the Brazilian North coast [34]. The processing enterprises are strategically located, have their own unloading ports, good operational and distribution structure, are connected to the electricity grid, and have their own generators, which guarantee the quality of the frozen products [34].

### 3.2. Output (triple bottom line—TBL)

**Fig 6:**
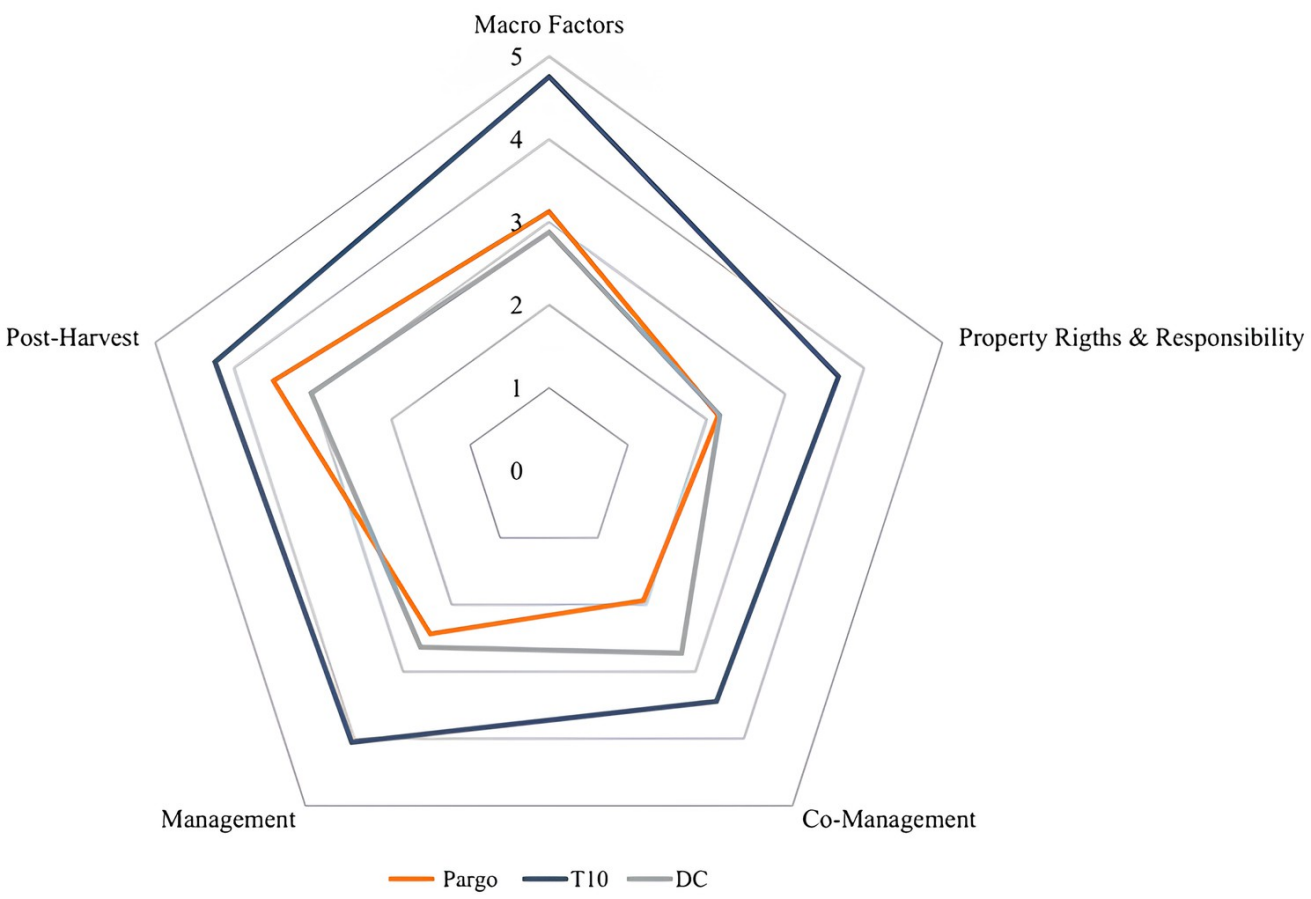
Comparison of the TBL *Output* scores for the large-scale artisanal snapper fishery *Lutjanus purpureus* (red), with the average of the top 10 world fisheries (T10—blue) and the with the 97 Developing Countries (DC—gray).

*Ecology* was the component that scored the lowest (2.3), followed by *Economics* (2.8) and *Community* (3.8) (Fig 6). The Ecology and Economy dimensions scored below the T10 and DC averages, however, *Community* was like the DC averages and lower than the T10 averages.

#### 3.2.1. Ecological indicator

**Fig 7:**
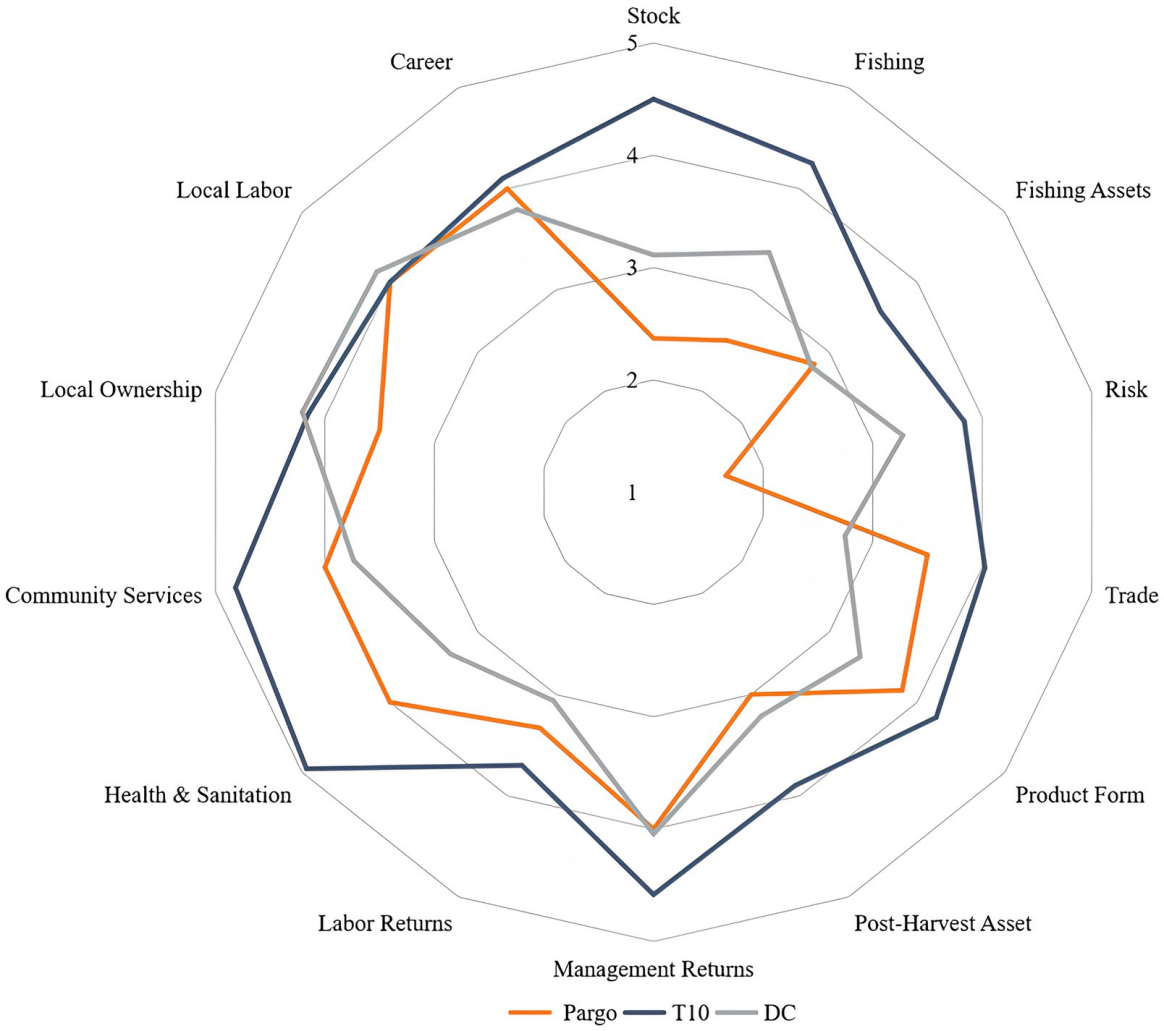
Average *Output* scores of the artisanal red snapper fishery *Lutjanus purpureus* (red) compared to the top 10 world fisheries (T10—blue) and to Developing Countries (DC—gray).

The ecological performance obtained the lowest score among the production averages (2.3). Compared to T10 and DC, the red snapper FPI averages are at unsatisfactory levels for a fishery considered sustainable (Fig 7). Results with averages below the global T10 and DC were also obtained in FPI studies for other Brazilian fisheries, such as brown shrimp *Penaeus subtilis* [34] and Acoupa weakfish *Cynoscion acoupa* [35].

One of the main reasons for this unsustainability is the average catch size of specimens (26 cm) [10], which is smaller than the established size of first sexual maturation, which occurs from a total length of 32 cm [10, 89]. Studies consider the possibility of growth overfishing, due to the reduction of the size of individuals, age and weight in fishing samplings [12, 86, 87, 90].

The possible overexploitation of the species contributed to the low scores for the metrics *Percentage of Overfished Stocks* (3.0), *Degree of Overfishing—Stock Status* (2.0), and *Declining*, *Stable*, *or Rebuilding Stock* (1.0), included in the *Ecologically Sustainable Fisheries* dimension. The percentage of stocks considered overfished reflects the extent to which overfishing has compromised the ability to generate sustainable livelihoods [24].

Direct quantitative information on the red snapper stock is still a gap in knowledge. The lack of systematized biological data is one of the main factors that make it difficult to assess stock health and may hide species that already face a situation of fishing vulnerability. Demersal species are more vulnerable to overexploitation due to their life history characteristics, such as long life span, slow growth and late sexual maturation [31, 91, 92].

Notably, changes in the age structure of the population have been reported, due to the high percentage of juveniles in landings and the decline in annual production and CPUE [10, 13], however, the fishing industry shows little concern with a scenario of declining production, since there is reluctance in the sector to accept management instructions based on scientific studies. Perhaps this opposition occurs because, even with the loss of market (USA and Europe), the sector still exports to less demanding markets.

Another important metric for ecological analysis that scored poorly in the snapper fishery system was *Illegal*, *Unregulated or Unreported Landings* (2.0). It assesses the proportion of landings from the managed stock using gear, areas, or methods that are considered illegal, are not reported, or are outside the regulatory framework [24]. Although the Normative Instruction 7 of 2004 of the Ministry of Environment, establishes standards for the capture of red snapper, even assigning a specific license, illegal vessels operating in the system are frequent and the quantity is unknown. It is assumed that there is almost double the number of licensed vessels acting clandestinely [13, 42]. These illegal practices pose serious risks to sustainability and are the main drivers of overfishing in Brazil [35, 92].

#### 3.2.2. Economical indicator

The economic indicator scored poorly (2.8) (Fig 6), with all included dimensions scoring below 4 and lower than the T10 and DC, even lower than in other Brazilian fisheries, such as the brown shrimp [34] and Acoupa weakfish fisheries [35]. The best scores were assigned to the *Product Form* (3.8), Trade (3.5) and *Post-Catch Performance* (3.0) dimensions. The *Risk* (1.6), *Fishing* (2.5) and *Fishing Assets* (2.8) dimensions were the most unsatisfactory in the analyses. These dimensions reflect the sources of risk in the fishery that may inhibit economic investment allocated to the fishery in addition to indicating the aspects that are being essential to generate sustainable income from landings [24].

The annual volatility of total revenue is a measure that explains the degree of economic risk in a fishery as well as the volatility of landings [24]. The results of the present work show that the red snapper fishery has high price volatility and economic risks that can cause future problems such as investment uncertainty. Directly, the context of risk is also reflected in the lack of reference points that guide investments in the snapper fishery. Stock assessments that could allow for specific forms of management such as global quotas are non-existent due to the lack of reliable production and effort data.

Despite the lack of specific guidelines, Brazil maintains subsidies that foster fishing activity. The program to reduce diesel oil costs through tax exemption lacks a systematic evaluation of stocks and fisheries. Studies categorize fuel subsidies as detrimental due to the stimulation of increased effort and the resulting contribution to overfishing [93] also impacting long-term social and economic benefits [94].

The main importer of red snapper from Brazil was the USA [55, 56]. However, the country has implemented the Seafood Import Monitoring Program (SIMP), which prevents the entry of illegal products or products of unsustainable ecological origin [95]. In this scenario, the snapper fishery, regarding all the aspects that make it unsustainable and without any optimistic projection of maintaining the activity, has been losing space in many important markets. The red snapper industry remains a sector that is not very open to market changes and, like the Brazilian brown shrimp industry, has looked to other, less demanding markets. However, this is not a good long-term strategy, as international markets are increasingly demanding eco-labeled seafood [96]. We suggest that the industry seek to conform to the standards of the Fishery Improvement Projects (FIPs) program, which are multi-stakeholder initiatives aimed at encouraging sustainable fisheries. In addition, complying with the measures already proposed in the red snapper Recovery Plan [13], adopting catch limits [73], catching individuals larger than the estimated L50 [10, 97] may generate positive impacts on the credibility of the activity and help the faster recovery of stocks.

#### 3.2.3. Community indicator

The Community indicator assesses the socioeconomic conditions that the fishery provides to the community [24]. In the present study, the average score (3.9) was close to the DC score (3.8) and below the T10 score (4.3).

Currently, the red snapper fleet employs approximately 2,500 people, considering only those workers who work directly inside the licensed vessels. The crew consists of up to 12 crewmembers per vessel, these being the master, professional fish cooler, vessel driver, and fishermen [42]. Crew remuneration differs according to the type of fishing gear used (‘*pargueira* line’ and ‘*manzuá’*). In vessels with ‘*manzuá’*, payment is made based on production, where the boat master is the best paid and holds 15%, driver 4%, professional fish cooler—responsible for gutting and cooling the fish—3.5% and 2% for each fisherman [13]. In longline fisheries, the payment is fixed between 11% and 15% for the master, 4.5% to 5% for the driver, 3% to 4% for the professional fish cooler, and fishermen are paid per kilogram of fish [13]. In addition, the fishermen have a labor contract, which guarantees them labor rights. In both cases, large-scale artisanal fishing for red snapper grants those involved a higher income than in other artisanal fisheries, offering a better quality of life, with access to education, health, and leisure. In fact, fishing provides those involved with good economic returns, taking into consideration that most fishermen have only a basic education, unlike workers in the processing and sales industries.

The life quality of Amazonian fishermen is traditionally discussed in its different approaches [98], however, the human perception of quality of life requires an interdisciplinary analysis, since it relies on a diverse set of knowledge to achieve a broad understanding of the subject of study [99]. Based on this assumption, quality of life studies involving industrial and artisanal fishermen, as well as indigenous people, ‘quilombolas’ (isolated traditional population descendant of African slaves), extractives, and riverbank dwellers, must respect the perception of the way of life existing in each group. In the case of red snapper fishermen, even admitting the subjectivity of the concept of quality of life, and considering the better incomes obtained by fishing, it is admitted that they have better conditions than local artisanal fishermen [12].

The results showed that the *Management Returns* for the red snapper fishery are good (4.0). This means that vessel owners, ship owners and masters have socioeconomic benefits by receiving most of the profits. On a hierarchical scale, *Labor Return* measures the fishermen’s earnings, opportunities, and social status. In this aspect, the dimension scored 3.3, meaning that the actors involved also have basic social access that small-scale artisanal fishers do not have [100].

For *Health and sanitation*, *Local Labor* and *Career* was assigned a score of 4. A socioeconomically successful fishery provides access to health and safety at work for its employees and generates stable employment in the long term. In the red snapper fishery, like other fisheries, many data needed to assess social and economic performance are lacking; information on prices, employment, effort, working conditions, and many other factors are not consistently collected for systematic cross-comparison [24, 25].

The large-scale artisanal fishery for red snapper is a fishing activity that contributes significantly to the Brazilian economy. This study highlights that the main positive aspects are related to community well-being, providing more equitable remuneration for those involved and thereby encouraging improved performance in work development and quality of life. However, it is important to note that these participants are often excluded from the decision-making process, lack representation in crucial meetings, and may be unaware of the level of vulnerability inherent in the fishing activity itself.

Although the FPI tool has limitations [31], the results were able to point out important problems that put the permanence of the activity and the maintenance of the species *L*. *purpureus* at risk, they are: (i) fishing of juveniles; (ii) illegal vessels; (iii) lack of collaboration of the fishing sector with science and (iv) unreliability of the data provided. All the above points make the red snapper fishery on the Amazon continental shelf socioecologically unsustainable in the long term.

Although the current Interministerial Ordinance No. 42/2018 is thorough in some aspects for the capture permission, the red snapper fishing activity is not strictly inspected, having as the only monitoring device of the fishing areas the PREPS, which only monitors boats that own and activate the equipment, which is not the case of illegal vessels operating in the Brazilian North and Northeast areas.

Although the red snapper productive sector has cooperated with academia and researchers for the elaboration of the Recovery Plan in 2018, it is still evident the reluctance of the sector to agree with what is proposed, as well as, to adopt sustainable fishing measures. Many information gaps about the activity are the result of ineffective management cycles that could be partially or completely remedied through more inclusive and adaptive management policies.

The present study is the first to provide a joint assessment of three interconnected sectors of the northern Brazilian red snapper fishery, showing, even with limited data, how the snapper fishery is developing and heading towards an uncertain future. Fisheries collapses seem to have become common for some world fisheries. Data indicate typical patterns emerging from analysis of catch series during the period preceding collapses: smooth collapse (33%), i.e. a long steady decline, erratic collapse (45%), i.e. a decline after several ups and downs, and plateau collapse (21%), i.e. a sudden decline after a relatively long and stable persistence of high level catches [101].

Fisheries like the red snapper fishery described in this paper exist because there is a profitable trade behind it that drives a whole value chain that perpetuates itself over time even with clear signs of depletion. There are only clandestine boats because there are industries that feed this trade based on a very profitable cumulative profit. The current underestimation—mainly by the fishing sector—of an ever-increasing fishing efficiency, associated with the lack of resilience of the red snapper stocks and the governmental mechanisms of fishing subsidies, will probably promote a collapse of the red snapper system in a relatively short time.

Proper quantification of effective fishing effort—especially within the industries that make the entire system dynamic viable—and the effects of environmental variability in the face of climate change, must be an important step, coupled with restoration of depleted stocks, if we really decide to bring the snapper fishery back to viability, and reduce impacts on the Amazon reef.

## 4. Conclusions

This is the first study in which Fishery Performance Indicators were used to measure the performance of the commercial red snapper fishery in Northern Brazil. The results indicate that the fishery is not sustainable, and that the continuity of the activity is at risk if immediate management measures are not taken. Ecological factors exhibited worse performance, mainly attributed to the potential risk of overfishing. To address this, it is strongly advised to implement monitoring programs focused on data collection to estimate both abundance and stock status indicators, offering insights into the ecological dynamics and aiding in the effective management of fisheries resources. However, the social factor was positive, but with points that can be improved, such as the inclusion of fishermen in decision-making issues in the fishery. A strengthening of the regulatory mechanisms, especially in an ecologically correct market system, associated with an efficient monitoring of effort and production should be applied, since the industries have improved their efficiency to guarantee the stability of their catches and revenues, despite the decrease in fish abundance.

## Supporting information

S1 Table Overview of the input and output data spreadsheet for research results analysis. (PDF)

S2 Table Overview of the input and output data spreadsheet for research results analysis. (PDF)
