# Correction: Caribbean red snapper fishing performance indicators in Brazilian amazon shelf: Is it the beginning of the end of a fishing system?

**DOI:** 10.1371/journal.pone.0306070

**Published:** 2024-06-25

**Authors:** Niedja Mescouto, Ualerson Iran Peixoto, Diego Gomes Trindade, Hanna Moura, Bianca Bentes

## Notice of Republication

An incorrect version of Fig 2 was published in error. This article was republished on June 14, 2024, to correct the error. Please download this article again to view the correct version.
