# Supplementary material for: Caribbean red snapper fishing performance indicators in Brazilian amazon shelf: Is it the beginning of the end of a fishing system?
Source: PLoS One. 2024 May 1;19(5):e0300820. doi: 10.1371/journal.pone.0300820 (PMC11062544; doi:10.1371/journal.pone.0300820)
Supplement: S1 Table — (PDF) [file pone.0300820.s001.pdf]

Component	Dimension	Metric	Quality	Score	Average by Dimension	Average by Category
**Macro Factors**	General Environmental Performance	Environmental Performance Index (EPI)	A	**3**	2	3,1
	Exogenous Environmental Factors	Diseases and pathogens	B	**5**	5	
		Natural disasters and catastrophes	B	**5**		
		Pollution crashes and accidents	B	5		
		Chronic pollution level (stock effects)	B	5		
		Level of chronic pollution (consumption effects)	B	5		
	Governance	Quality of governance	A	3	3	
		Governance responsiveness	A	3		
	Economic Conditions	Index of economic freedom	A	2	2,5	
		Gross Domestic Product (GDP) per capita	A	3		
**Property Rights & Responsibility**	Fishing Access Rights	Proportion of fisheries managed under limited access	B	4	3,3	2,1
		Transferability Index	A	3		
		Security Index	A	4		
		Durability Index	A	5		
		Flexibility Index	A	2		
		Exclusivity Index	B	2		
	Fishing Rights	Proportion of fisheries managed under limited access	A	1	1	
		Transferability Index	A	not applied		
		Security Index	B	not applied		
		Durability Index	B	not applied		
		Flexibility Index	B	not applied		
		Exclusivity Index	A	not applied		
**Co-Management**	Collective Action	Proportion of fishermen in industry organizations	B	2	2	2
		Influence of Fishing Organization on Management and Access	B	3		
		Influence of Industry Organization on Business and Marketing	B	1		

	Participation	Days in stakeholder meetings	B	2	2	
		Industry financial support for management	B	2		
	Community	Leadership	B	3	2,5	
		Social Cohesion	B	2		
	Gender	Influence of business management	A	1	1,5	
		Influence of resource management	A	3		
		Labor Participation in the Harvest Sector	A	1		
		Labor Participation in the Post-Harvest Sector	A	1		
**Management**	Management Entries	Management Expenses Compared To The Value Of Fishing	B	3	3,5	3,1
		Application Capacity	B	2		
		Management Jurisdiction	B	5		
		Subsidy Level	B	4		
	Data	Data Availability	B	3	3	
		Data Analysis	B	3		
	Management Methods	MPAs and Sanctuaries	A	1	3	
		Spatial Management	B	4		
		Fishing Mortality Limits	B	4		
**Post-Capture**	Markets and Market Institutions	Landing Price System	C	1	3,3	3,6
		Availability of price and quantity information on departure from the ship	C	3		
		Number of Buyers	A	3		
		Degree of Vertical Integration	B	4		
		Rate Level	B	5		
		Level of Non-tariff Barriers	B	4		
	Infrastructure	International Freight Service	C	4	4	
		Road Quality Index	B	4		
		Technology Adoption	B	3		
		Extension Service	A	3		
		Reliability of Utilities / Electricity	B	5		
		Access to Ice and Refrigeration	A	5		
