# Supplementary material for: Caribbean red snapper fishing performance indicators in Brazilian amazon shelf: Is it the beginning of the end of a fishing system?
Source: PLoS One. 2024 May 1;19(5):e0300820. doi: 10.1371/journal.pone.0300820 (PMC11062544; doi:10.1371/journal.pone.0300820)
Supplement: S2 Table — (PDF) [file pone.0300820.s002.pdf]

| Indicator | Dimension | Metric | Quality | Score | Average by Dimension | Average by Category |
|---|---|---|---|---|---|---|
| **Ecology** | Ecologically Sustainable Fisheries | Percentage of Stocks Overfished | A | 3 | 2,3 | 2,3 |
| | | Degree of Overfishing-Stock Status | A | 2 | | |
| | | Stock Declining, Stable or Rebuilding - Stock Dynamics | A | 1 | | |
| | | Regulatory Mortality | A | 4 | | |
| | | Selectivity | A | 3 | | |
| | | Illegal, Unregulated or Unreported Landings | B | 2 | | |
| | | Status of Critical Habitat | A | 3 | | |
| | | Proportion of Harvest with a 3rd Party Certification | A | 1 | | |
| **Economics** | Fishing | Landings Level | C | 2 | 2,5 | 2,8 |
| | | Excess Capacity | B | 3 | | |
| | | Season Length | A | 3 | | |
| | | Ex-vessel Price Compared to Historic High | B | 2 | | |
| | Harvest Assets | Ratio of Asset Value to Gross Earnings | A | 2 | 2,8 | |
| | | Total Revenue Compared to Historic High | A | 2 | | |
| | | Asset (Permit, Quota, etc...) Value Compared to Historic High | A | 2 | | |
| | | Borrowing Rate Compared to Risk-free Rate | B | 4 | | |
| | | Source of Capital | B | 3 | | |
| | | Functionality of Harvest Capital | A | 4 | | |
| | Risk | Annual Total Revenue Volatility | A | 2 | 1,6 | |
| | | Annual Landings Volatility | B | 2 | | |
| | | Intra-annual Landings Volatility | B | 2 | | |

| | | | | | | |
|---|---|---|---|---|---|---|
| | | Annual Price Volatility | B | 1 | | |
| | | Intra-annual Price Volatility | B | 1 | | |
| | | Spatial Price Volatility | B | 2 | | |
| | Trade | Final Market Wealth | A | 3 | 3,5 | |
| | | International Trade | A | 4 | | |
| | | Wholesale Price Compared to Similar Products | B | 2 | | |
| | | Capacity of Firms to Export to the US & EU | B | 5 | | |
| | Product Form | Final Market Use | A | 4 | 3,8 | |
| | | Ex-vessel to Wholesale Marketing Margins | B | 3 | | |
| | | Processing Yield | B | 4 | | |
| | | Shrink | A | 5 | | |
| | | Capacity Utilization Rate | B | 5 | | |
| | | Product Improvement | B | 2 | | |
| | Post-Harvest Asset Performance | Borrowing Rate Compared to Risk-free Rate | A | 4 | 3,0 | |
| | | Source of Capital | B | 3 | | |
| | | Age of Facilities | B | 2 | | |
| **Community** | Managerial Returns | Earnings Compared to Regional Average Earnings | B | 5 | 4,0 | 3,8 |
| | | Owner/Permit Holder/Captain Wages Compared to Non-fishery Wages | B | 5 | | |
| | | Social Standing of Boat Owners and Permit Holders | B | 3 | | |
| | | Earnings Compared to Regional Average Earnings | A | 5 | | |
| | | Manager Wages Compared to Non-fishery Wages | B | 3 | | |
| | | Social Standing of Processing Managers | B | 3 | | |
| | Labor Returns | Earnings Compared to Regional Average Earnings | B | 4 | 3,3 | |

| | | | | | | |
|---|---|---|---|---|---|---|
| | | Crew Wages Compared to Non-fishery Wages | A | 4 | | |
| | | Social Standing of Crew | B | 3 | | |
| | | Earnings Compared to Regional Average Earnings | B | 3 | | |
| | | Worker Wages Compared to Non-fishery Wages | C | 3 | | |
| | | Social Standing of Processing Workers | B | 3 | | |
| | Health & Sanitation | Harvest Safety | B | 5 | 4,0 | |
| | | Access to Health Care | B | 4 | | |
| | | Access to Health Care | B | 3 | | |
| | | Sanitation | A | 5 | | |
| | | Access to Health Care | B | 4 | | |
| | | Access to Health Care | B | 3 | | |
| | Community Services | Contestability & Legal Challenges | B | 4 | 4,0 | |
| | | Education Access | B | 5 | | |
| | | Education Access | B | 3 | | |
| | | Regional Support Businesses | B | 4 | | |
| | | Education Access | B | 5 | | |
| | | Education Access | B | 3 | | |
| | Local Ownership | Proportion of Nonresident Employment | B | 4 | 3,5 | |
| | | Nonresident Ownership of Processing Capacity | B | 3 | | |
| | Local Labor | Proportion of Nonresident Employment | B | 4 | 4,0 | |
| | | Proportion of Nonresident Employment | B | 4 | | |
| | Career | Crew Experience | B | 5 | 4,0 | |
| | | Age Structure of Harvesters | B | 4 | | |
| | | Worker Experience | B | 3 | | |
